# Supplementary material for: Recommendations for evaluating photoplethysmography-based algorithms for blood pressure assessment
Source: Commun Med (Lond). 2024 Jul 12;4:140. doi: 10.1038/s43856-024-00555-2 (PMC11245506; doi:10.1038/s43856-024-00555-2)
Supplement: Supplementary file 2 — Description of Additional Supplementary Files [file 43856_2024_555_MOESM2_ESM.docx]

**Legend for Supplementary Data 1**

**Supplementary Data 1.** Analysis of Evaluation Measures in Single and Multi-Site PPG-Based Blood Pressure Models from Recent Decade.

**Description:** This supplementary file presents detailed data gathered from a variety of studies on photoplethysmography (PPG)-based blood pressure models. It encompasses a thorough review of 25 single-site PPG models and 13 multi-site PPG models. The dataset features an exhaustive list of studies analyzed, emphasizing the evaluation measures used and the number of participants involved in each study.

**Contents:**

- **Year**: The publication year of the study.
- **Participant**: The number of participants involved in the study.
- **ME ± SDE (Mean Error ± Standard Deviation of Error)**: Indicates whether the study reported mean error and standard deviation of error. 'Yes' signifies reported, and 'No' signifies not reported.
- **MAE (Mean Absolute Error)**: Indicates whether mean absolute error was reported. 'Yes' for reported, 'No' for not.
- **RMSE (Root Mean Square Error)**: Indicates the reporting of root mean square error.
- **Pearson Correlation Coefficient**: Indicates if the Pearson correlation coefficient was calculated and reported.
- **Number over 5, 10, 15**: Refers to the number of studies that reported absolute errors within 5, 10, and 15 mmHg.
- **Band Altman Plot**: Indicates whether the Bland-Altman plot was used to assess agreement between gold standard and PPG-based blood pressure in the study.

**Purpose**: The data compiled in this Excel file supports the analysis and discussion of the effectiveness and commonality of various evaluation measures used in PPG-based blood pressure modeling. It specifically helps illustrate trends such as the frequent or infrequent use of certain statistical measures across the reviewed studies.

**File Format**: Excel spreadsheet (.xlsx)
